# The potential therapeutic effect of melatonin on human ovarian cancer by inhibition of invasion and migration of cancer stem cells

**DOI:** 10.1038/s41598-017-16940-y

**Published:** 2017-12-06

**Authors:** Maryam Akbarzadeh, Ali Akbar Movassaghpour, Hossein Ghanbari, Maryam Kheirandish, Nazila Fathi Maroufi, Reza Rahbarghazi, Mohammad Nouri, Nasser Samadi

**Affiliations:** 1 Stem Cell And Regenerative Medicine Institute, Tabriz University of Medical Sciences, Tabriz, Iran; 2 Biotechnology Research Center, Tabriz University of Medical Sciences, Tabriz, Iran; 3 Hematology and Oncology Research Center, Tabriz University of Medical Sciences, Tabriz, Iran; 4 Department of Medical Nanotechnology, School of Advanced Technologies in Medicine, Tehran University of Medical Sciences, Tehran, Iran; 5 Department of Immunology Blood Transfusion Research Center, High Institute for Research and Education in Transfusion Medicine, Tehran, Iran; 6 Department of Biochemistry and Clinical Laboratories, School of Medicine, Tabriz University of Medical Sciences, Tabriz, Iran; 7 Stem Cell Research Center, Tabriz University of Medical Sciences, Tabriz, Iran

## Abstract

There is an urgent need to identify targeting molecules to control invasion and metastasis in cancer patients. We first isolated cancer stem cells (CSCs) from SKOV3 ovarian cancer cells and then investigated the role of melatonin in invasiveness and migration of CSCs compared to SKOV3 cells. The proportion of CSCs in SKOV3 cells was as low as 1.28% with overexpression of both CD133 and CD44. The ability of spheroid formation along with SOX2 overexpression revealed a high self-renewal potential in isolated cells. Melatonin (3.4 mM) inhibited proliferation of CSCs by 23% which was confirmed by a marked decrease in protein expression of Ki67, as a proliferation marker. Applying luzindole, a melatonin receptor 1, 2 inhibitor, partially abolished anti-proliferative effect of melatonin. Melatonin also decreased Epithelial mesenchymal transition (EMT) related gene expressions including ZEB1, ZEB2, snail and vimentin with increase in E-cadherin as a negative EMT regulator. Incubation of CSCs with melatonin showed a marked decrease in matrix metalloproteinase 9 (MMP9) expression and activity. Melatonin also inhibited CSCs migration in a partially receptor dependent and PI3k and MAPK independent manner. Melatonin can be considered as an important adjuvant to control invasion and metastasis especially in patients with high melatonin receptor expression.

## Introduction

Ovarian cancer is the fifth most common gynecological malignancy. Most of the patients are diagnosed in advanced stages. Despite conventional treatments such as surgery and platinum-based chemotherapy, tumor recurrence can be observed in the most patients. Therefore, developing effective treatment strategies can be critical in ovarian cancer therapy^1^.

More recently, increasing evidence represented the existence of highly tumorigenic cells with stem cell properties within the various tumor microenvironments including ovarian cancer^2^. Moreover, these stem cells are found in cancer cell lines which were previously thought to be homogenous^3^. The important features of this rare population are its ability to self-renewal, clonogenicity and multi-differentiation capacities^4^. Cancer stem cells (CSCs) can be isolated and characterized by specific surface markers such as CD133, CD44, and CD117^2^. A growing body of evidence also declares that CSCs and embryonic stem cells share common stemness molecules including SOX2, Nanog and Oct4^5^. CSCs are critically contributed to tumor initiation, metastasis, relapse and resistance to chemotherapy^2^. Therefore, targeting these cells can be considered as a novel strategy for efficient cancer therapy.

Melatonin is a natural hormone that synthesized and secreted by the pineal gland as well as other organs such as retina, skin, ovary, intestine and testes^6^. A large number of studies have identified that melatonin plays a key role in regulation of many biological processes including circadian rhythms, reproduction, hormone secretion and immunomodulation^7^. In addition to the main physiological roles, melatonin displays oncostatic and tumor-inhibitory effects with no side effect on pharmacologic concentrations in various cancers thereby there is a lot of interest for applying this molecule in cancer therapy^8,9^. Functions of melatonin are mediated by receptor-dependent or –independent mechanisms^9,10^. The most functional cell surface receptors of melatonin are MT1 and MT2 that belong to the G-protein coupled receptor family^9^. Activation of MT1 or MT2 inhibits cAMP production and mitogen activated protein kinase (MAPK) cascade as well as PI3K-dependent pathways^11,12^. Moreover, melatonin passes through the cell membrane, inhibits calmodulin and induces detoxification by radical scavenging abilities. Inhibition of calmodulin results in the reduction of cAMP accumulation and related signaling pathways^10^. Since several signaling pathways can produce the same effect, it is challenging to find out whether these reactions are mediated via receptors. To date, very few studies have investigated the effects of melatonin and underlying mechanisms on CSCs. It has been reported that melatonin inhibits self-renewal and related signaling pathways of glioma cancer stem cells^6^. The effects of melatonin on viability, invasiveness and metastasis in breast CSCs have also been postulated through regulation of epithelial-mesenchymal transition (EMT)^13^.

In this study, we first isolated CSCs from SKOV3 ovarian cancer cell line, and determined the stemness and self-renewal ability of these cells through both flow cytometry analysis for cell specific markers including CD133, CD44 and SOX2, as well as spheroid formation assay. Then, we demonstrated that melatonin inhibited proliferation and migration of CSCs through modulation of PI3K and MAPK signaling pathways in both receptor-dependent and independent manners. The effects of melatonin on invasion properties of CSCs were determined by MMP-2 and MMP-9 expression and activity panels. To study the impact of melatonin on EMT process, we measured key gene expression levels that are involved in this phenotype including ZEB1, ZEB2, snail, vimentin and E-cadherin. Proliferation, invasion and migration of CSCs are markedly higher than those in non-stem cell ovarian cancer cells. Our results suggest that melatonin can be considered as an important modulator to suppress tumor progression via targeting CSCs proliferation, migration and invasion especially in the patients with high melatonin receptor expression.

## Results

### Isolation and characterization of human ovarian cancer stem cells

**Figure 1:**
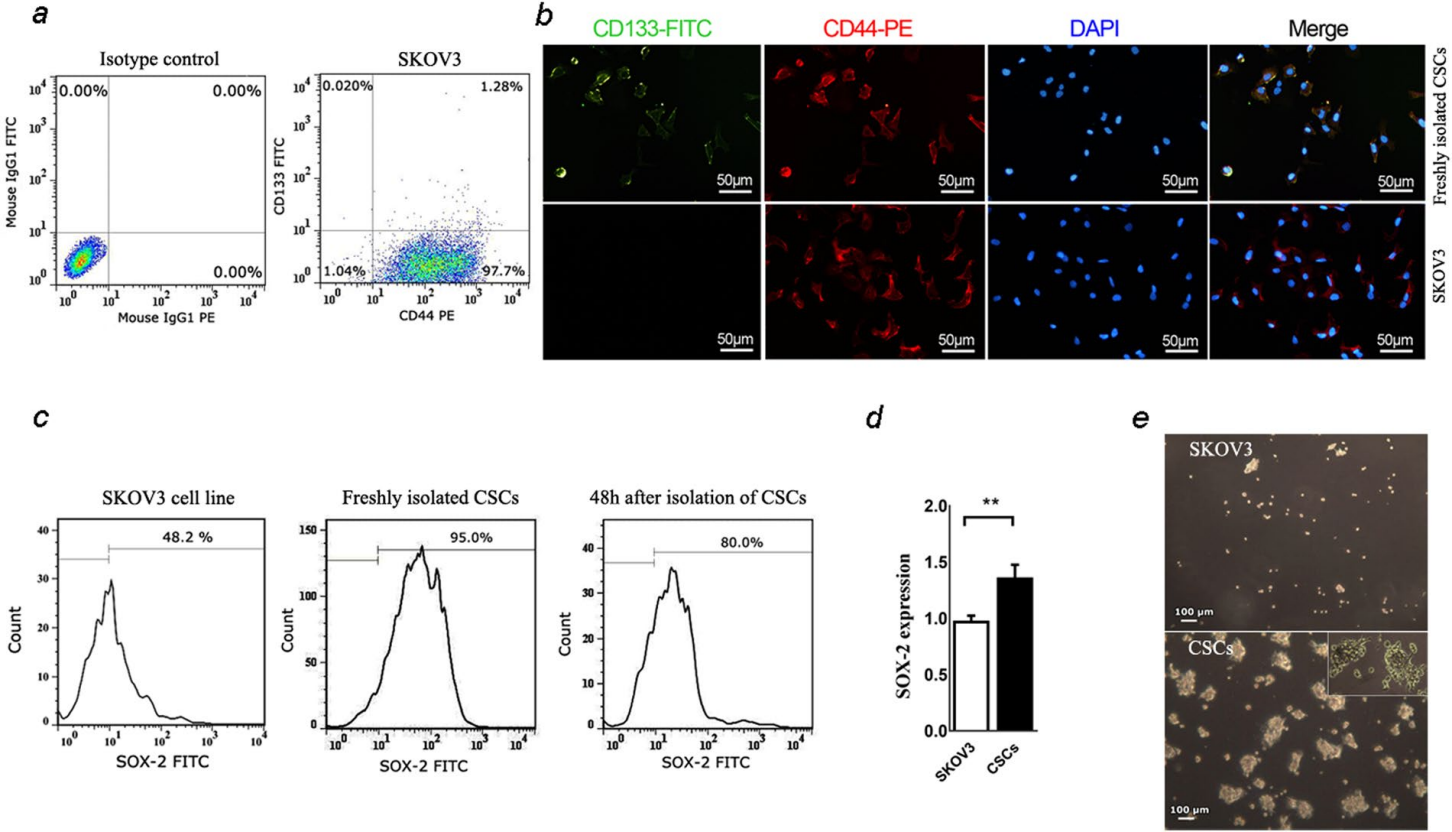
Cancer stem cells (CSCs) isolation and characterization from human ovarian SKOV3 cell line. (**a**) SKOV3 cells were labeled with FITC-conjugated anti-CD133 and PE-conjugated anti-CD44 antibodies, and analyzed by a flow cytometer as described in Materials and methods. (**b**) Immunofluorescence staining of CD133 and CD44 in CSCs and SKOV3 cell line. Cells were directly labeled using FITC-conjugated anti-CD133 (green) and PE-conjugated anti-CD44 (red) antibodies; nuclei were stained blue with 4′,6-diamidino-2-phenylindole (DAPI). (**c**) SOX2 protein expression was stained using FITC-conjugated anti-SOX2 and analyzed by Flow cytometry in SKOV3 cells, immediately after isolation and after 48 h incubation in medium supplemented by 2% fetal bovine serum (FBS). (**d**) SOX2 mRNA expression in CSCs was carried out by real-time RT-PCR and compared with that expression in SKOV3 cells. Data are expressed as mean ± SD of three independent experiments. Statistically significant differences are indicated as *p < 0.05. (**e**) Cells (3 × 104) were cultured in serum free media supplemented with 10 µM epidermal growth factor (EGF) and 10 µM basic fibroblast growth factor (bFGF) in non-treated six-well plates for seven days and spheroid formation was visualized by light microscopy in both CSCs and SKOV3 cells.

Identification of CSCs in SKOV3 cell line was determined based on the expression of both CD133 and CD44 markers (Fig. 1a). Flow cytometry analysis indicated that 97.3 ± 0.48% of the SKOV3 cell line expressed CD44. However, only 1.2 ± 0.25% of the cells were double positive for both CD133 and CD44 markers in SKOV3 cell line (Fig. 1a). In order to isolate CSCs from SKOV3 cell population, we performed magnetic-activated cell sorting (MACS) analysis using specific antibodies to collect CD133 positive cells. Immunofluorescence staining on isolated cells demonstrated that the majority of the cells were double positive for CD133 and CD44 markers compared to SKOV3 cells (Fig. 1b). To confirm the stemness properties of the isolated cells, SOX2 expression was also detected in these cells in both mRNA and protein levels (Fig. 1c,d). Our results revealed that majority of the cells expressed SOX2 protein in freshly MACS-isolated cells (95.15 ± 2.67%), which was confirmed by real-time RT PCR (Fig. 1d). Further detection of SOX2 expression after 48 h incubation of isolated CSCs showed no marked evidence of EMT process in these cells (Fig. 1c). The ability of CSCs for spheroid formation was examined by incubation of isolated cells in serum-free medium containing EGF and bFGF for 7 days. Spheroid formation assay showed a significant organization of spheroids which indicated self-renewal ability of isolated CSCs (Fig. 1e).

### Effects of melatonin on proliferation and stemness properties of CSCs

**Figure 2:**
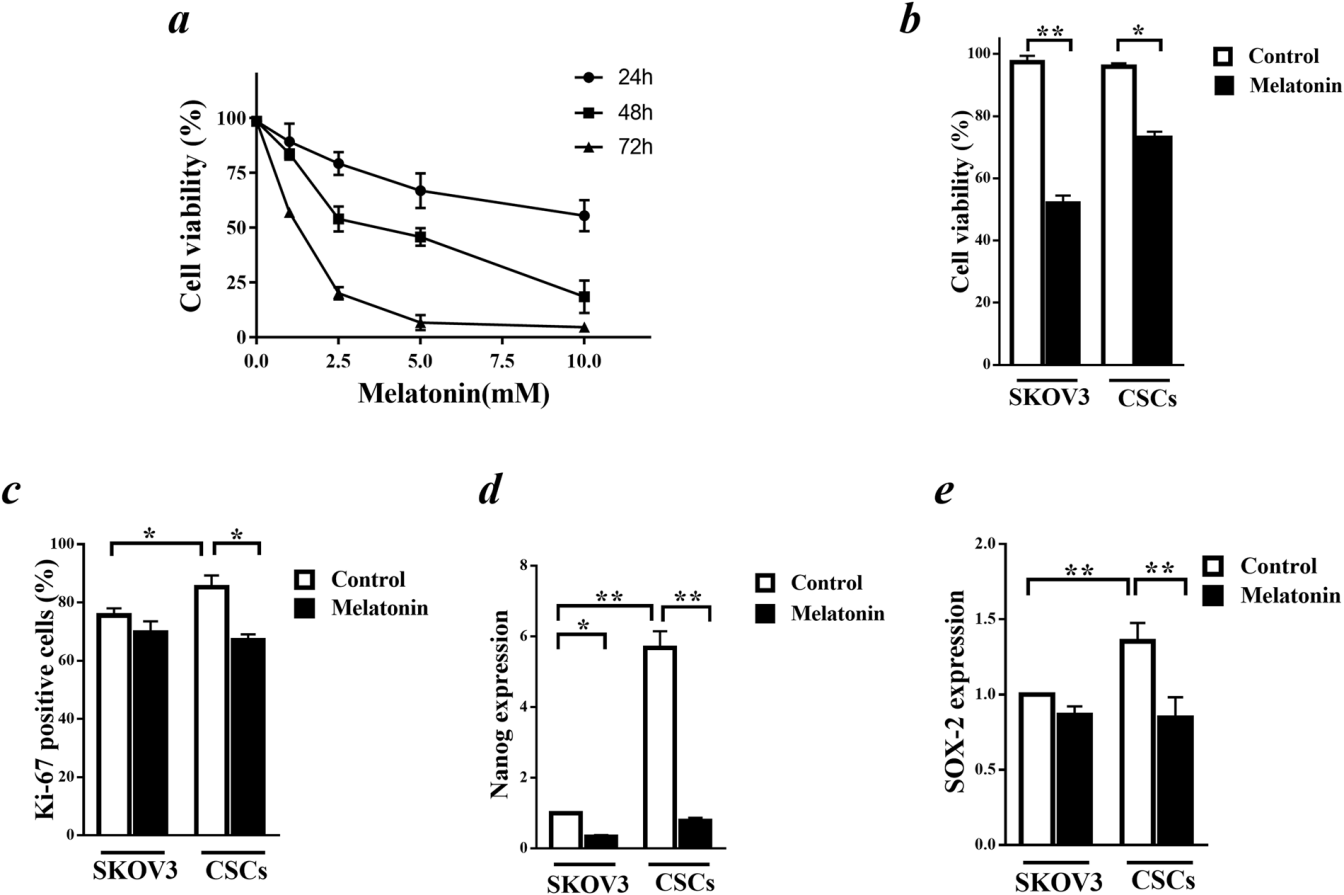
Melatonin effects on proliferation and stemness properties of cancer stem cells (CSCs). (**a**) Inhibitory effects of melatonin on viability of SKOV3 cells. (**b**) The effects of melatonin (IC50 value = 3.4 mM) on viability of CSCs and SKOV3 cells were determined after 48 h incubation. (**c**) The protein expression of Ki67 proliferation marker was determined after incubation of the cells with melatonin (3.4 mM) for 48 h in both CSCs and SKOV3 cells by applying FITC conjugated anti-Ki67 antibody and analyzed by flow cytometry. (**d**) Nanog mRNA expression in CSCs was carried out by real-time RT-PCR and compared with that expression in SKOV3 cells after incubation of the cells with melatonin (3.4 mM) for 48 h. (**e**) The role of melatonin in SOX2 stemness marker expression was evaluated after incubation of the cells with melatonin (3.4 mM) for 48 h using real-time RT PCR analysis Results are presented as mean ± SD of three independent experiments. Statistically significant differences are indicated as *p < 0.05 and **p < 0.01.

To determine IC50 value for melatonin, MTT assay was performed on SKOV3 cells at different time points. The growth inhibition of the cells after incubation with melatonin showed dose and time dependent pattern (Fig. 2a). IC50 of melatonin was 3.4 mM after 48 h incubation which was applied to subsequent experiments (Fig. 2a). Then, we investigated whether melatonin changes viability of isolated CSCs. Incubation of CSCs with melatonin for 48 h revealed almost a two fold increase in IC50 value (6.3 mM) in these cells. Melatonin (3.4 mM) decreased the viability of CSCs only 23% (Fig. 2b). To further assess whether melatonin affect cell proliferation, we investigated the expression of Ki67, a nuclear protein related to cellular proliferation using flow cytometry (Fig. 2c). Our data revealed that Ki67 was markedly more expressed in CSCs than SKOV3 cells (p < 0.05). In addition, treatment with melatonin significantly decreased Ki67 level in CSCs (up to 67.07%) compared to non-treated cells (up to 85.23%). There was no significant change in Ki67 expression level by applying melatonin in SKOV3 cells. Nanog and SOX2 expressions as key markers of stemness were also measured by real-time RT PCR (Fig. 2d,e). Nanog mRNA expression level revealed almost six fold increase in CSCs compared to SKOV3 cells which was completely diminished after 48 h incubation with melatonin (3.4 mM) (Fig. 2d). SOX2 mRNA level in CSCs was also significantly higher in CSCs than that level in SKOV3 cells (p < 0.05) which was also decreased after incubation with melatonin (p < 0.05) (Fig. 2e). These results indicated that melatonin reduced proliferation and stemness states in CSCs through inhibition of proliferation marker, Ki67, and stemness markers, Nanog and SOX2 expressions.

### The role of melatonin receptors in CSCs proliferation

**Figure 3:**
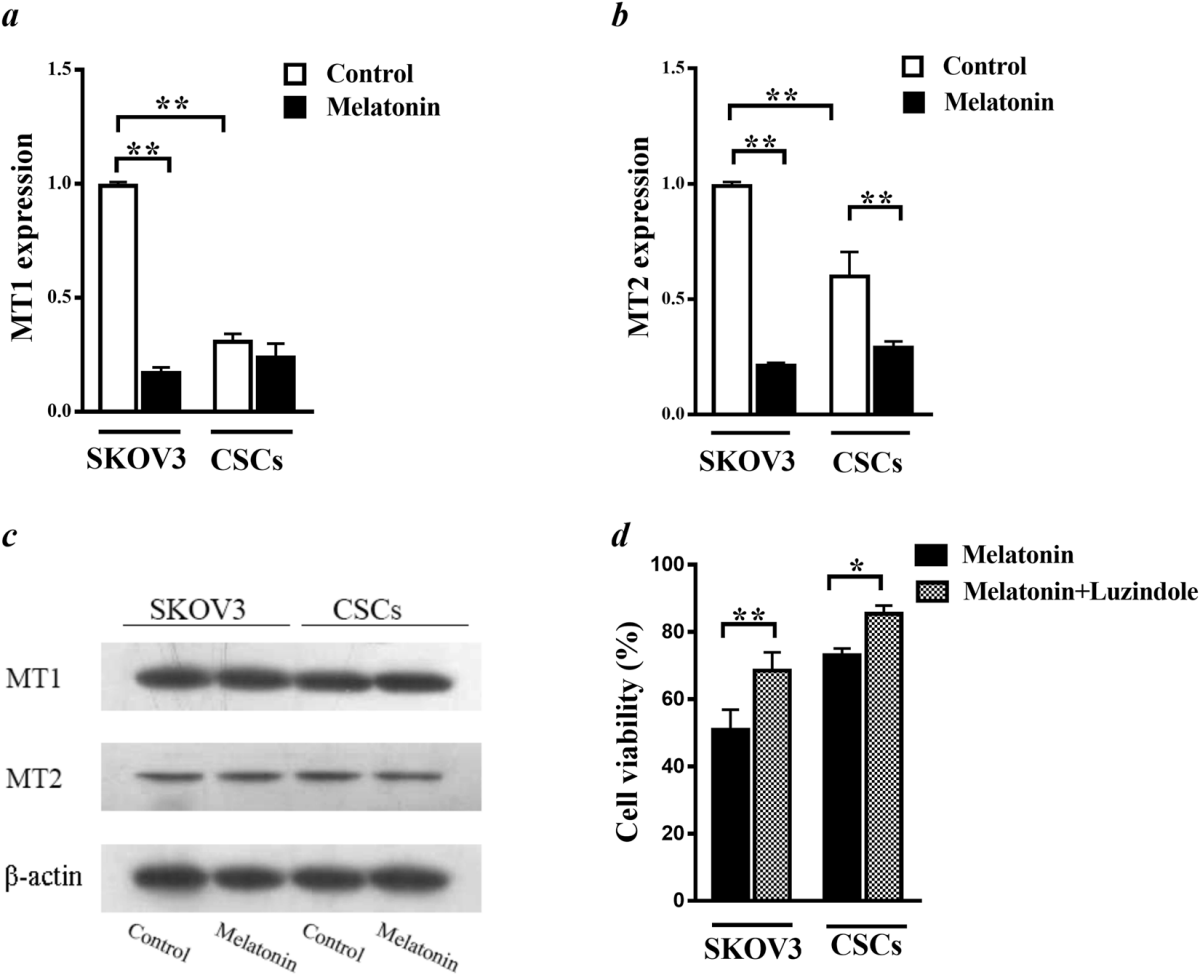
Effects of melatonin on MT1 and MT2 melatonin receptors in cancer stem cells (CSCs) and SKOV3 cells. MT1 (**a**) and MT2 (**b**) mRNA expression levels were determined by real-time RT PCR after incubation of both CSCs and SKOV3 cells after with and without melatonin (3.4 mM) for 48 h. Relative changes in gene expression levels were determined using the Pfaffl method. Data, normalized against GAPDH, are presented as fold change from the control. (**c**) SKOV3 and isolated CSCs were treated with the 3.4 mM melatonin for 48 hr. The cell lysates were prepared and used for Western blot with MT1, MT2 and b-actin antibodies. (**d**) The role of melatonin receptors on melatonin–induced changing in cell viability was evaluated after pretreatment of the cells with a pan melatonin receptor inhibitor (luzindole) and then incubation of the cells with melatonin (3.4 mM) for 48 h using MTT assay. All experiments were performed in triplicate and data were expressed as mean ± SD. *p < 0.05 and **p < 0.01.

First, we performed real-time RT PCR to evaluate the effect of melatonin on expression of melatonin receptors in CSCs versus SKOV3 cells. Our results showed that both MT1 and MT2 were expressed in both CSCs and non-CSCs with a higher expression level of MT1 receptor (Fig. 3a–c). Incubation of the cells with 3.4 mM melatonin was able to significantly decrease MT1 mRNA levels in SKOV3 but not in CSCs (Fig. 3a). However, there was a marked decrease in MT2 mRNA level in both CSCs and SKOV3 cells when we incubated the cells with 3.4 mM of melatonin for 48 h (Fig. 3b). To investigate the effect of melatonin on the protein expression levels of melatonin receptors, we applied western blot analysis in the presence and absence of melatonin in both CSCs and SKOV3 cells. Our results revealed a significantly higher MT1 protein expression compared to MT2 receptor in both SKOV3 cells and CSCs. Applying melatonin did not show any significant effect on the protein level of these proteins (Fig. 3c) (Supplementary information). Pre-incubation of the cells with luzindole (10 µM), an antagonist of MT1 and MT2 receptors revealed an increase in cell viability from 73.1 ± 2.1 to 85.3 ± 2.5 in CSCs and 50.86 ± 6 to 68.12 ± 5.5 in SKOV3 cells (Fig. 3d). Our data suggested that the function of melatonin in SKOV3 and CSCs was partially mediated by MT1 and MT2 receptors.

### The effects of melatonin on invasiveness of CSCs

**Figure 4:**
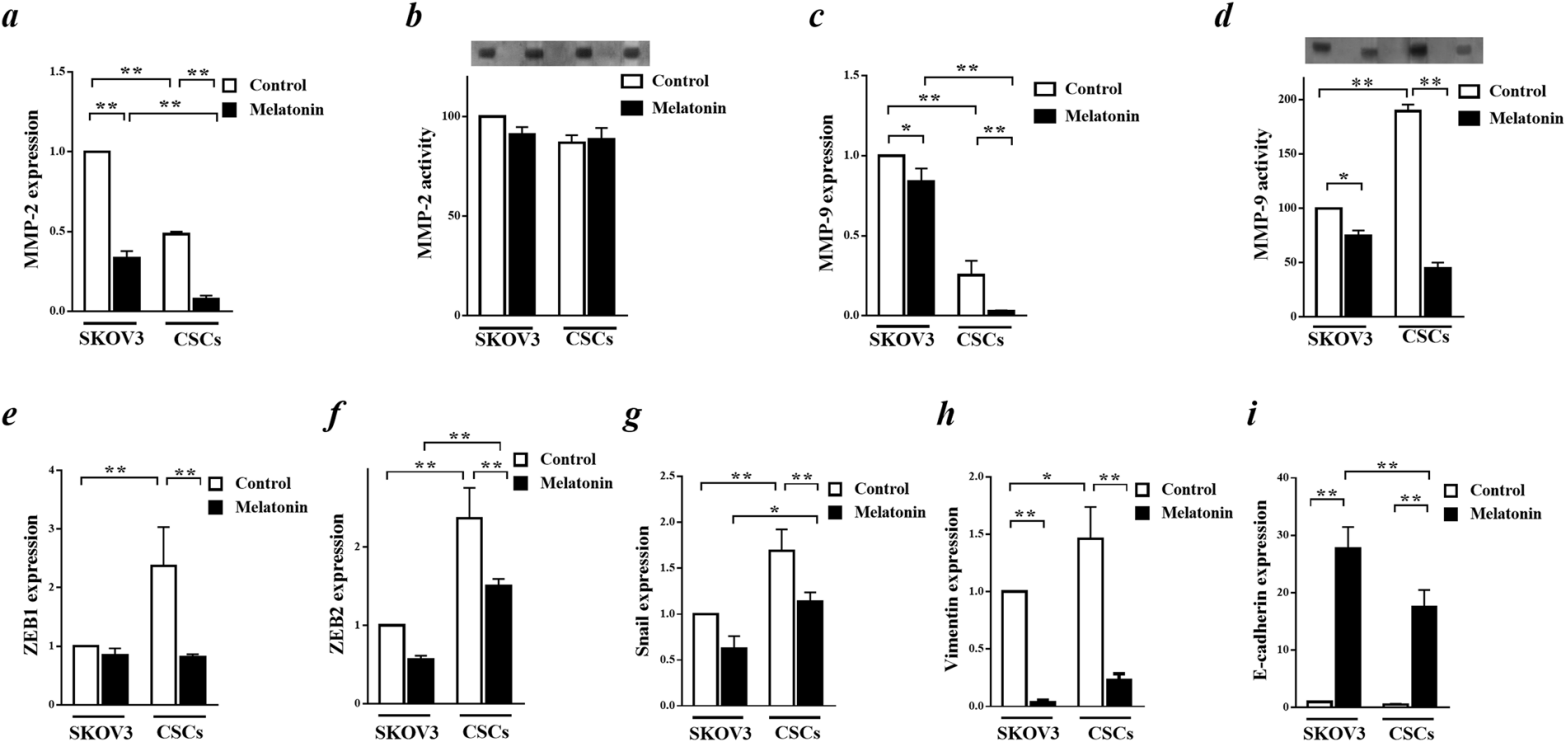
The role of melatonin in invasion activity of cancer stem cells (CSCs). (**a**) MMP-2 mRNA expression levels were determined by real-time RT PCR after 48 h incubation of both CSCs and SKOV3 cells with melatonin (3.4 mM). (**b**) Equal amounts of extracted protein following incubation with and without melatonin (3.4 mM) for 48 h were subjected to gelatin zymography to determine MMP-2 activity. (**c**) The role of melatonin in MMP-9 expression level was evaluated after incubation of the cells with melatonin (3.4 mM) for 48 h using real-time RT PCR analysis. (**d**) MMP-9 activity was determined by gelatin zymography after incubation with or without 3.4 mM melatonin for 48 h. (**e–i**) mRNA levels of ZEB1, ZEB2, Snail, vimentin and E-cadherin quantified by real-time RT PCR analysis. Relative changes in gene expression levels were determined using the Pfaffl method. Data, normalized against GAPDH, were presented as fold change from the control. All experiments were performed in triplicate and data were expressed as mean ± SD. *p < 0.05 and **p < 0.01.

To determine the effect of melatonin on invasiveness of CSCs, we evaluated the expression of MMP-2 and MMP-9 in both mRNA and activity levels (Fig. 4a–d). Zymography analysis revealed a significantly higher MMP-9 activity in CSCs compared to SKOV3 cells (Fig. 4d). There was no significant change in MMP-2 activity level between both cells (Fig. 4b). Incubation of the cells with melatonin caused a marked decrease in MMP9 activity without any significant change in MMP2 activity (Fig. 4b,d). Our results from real time RT-PCR showed a lower mRNA expression levels for both MMP2 and MMP9 in CSCs compared to SKOV3 cells. Applying melatonin decreased MMP-2 and MMP-9 mRNA expression levels in both CSCs and SKOV3 cells, however, maximum effect was observed in MMP-2 expression in CSCs (Fig. 4a,c).

In order to better understand the role of melatonin treatment on invasion, we investigated the expression of the genes that are involved in EMT including ZEB1, ZEB2, snail, vimentin and E-cadherin. EMT and invasion associated genes, vimentin, ZEB1, ZEB2, snail, were highly expressed in CSCs compared to SKOV3 cells (Fig. 4e–i). Incubation of the cells with melatonin (3.4 mM) for 48 h decreased the expression of these genes in both CSCs and SKOV3 (Fig. 4e–h). Maximum effect of melatonin was detected in vimentin expression level (Fig. 4h). E-cadherin, a negative regulator of EMT process, showed a marked increase under melatonin treatment in both CSCs and SKOV3 cells (p < 0.05) with a maximum increase in SKOV3 cells (Fig. 4i). Melatonin was able to decrease EMT and invasion properties of CSCs with reduction in MMP-9 activity and down regulation of EMT associated genes.

### Effects of melatonin on migration of CSCs

**Figure 5:**
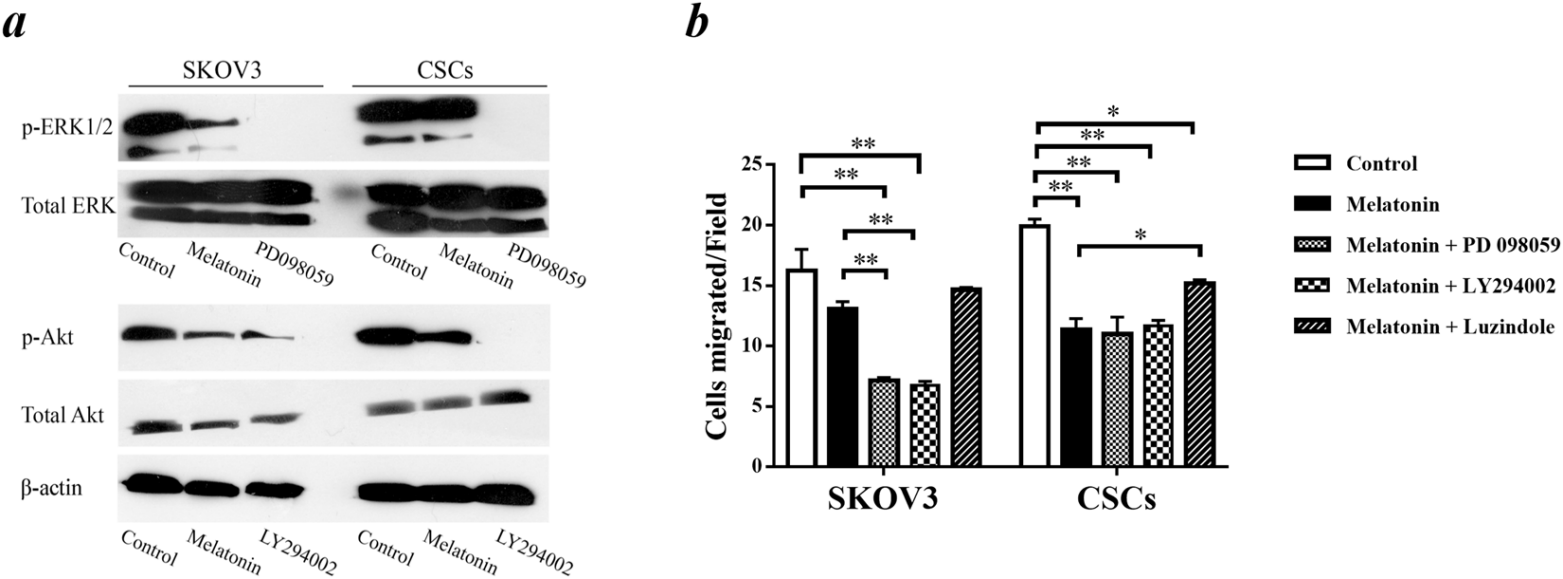
Effects of melatonin on migration ability in ovarian cancer stem cells (CSCs) and SKOV3 cells. (**a**) SKOV3 and isolated CSCs were treated with the 3.4 mM melatonin for 48 hr. The cell lysates were prepared and used for Western blot with p-ERK1/2, total ERK, p-Akt, total Akt and b-actin antibodies. (**b**) Cells were pretreated with MAPK inhibitor (PD98059) (40 µM), PI3K inhibitor (LY294002) (20 µM) and Melatonin receptor antagonist (luzindole) (10 µM) for 1 h and then incubated with melatonin (3.4 mM) for 48 h. Migrated cells in basal media (RPMI 1640 with 2% FBS) was compared to migration in the presence of melatonin with or without inhibitors. All experiments were performed in triplicate and data were expressed as mean ± SD. *p < 0.05 and **p < 0.01.

To investigate the effect of melatonin on the migration and underlying mechanisms we examined the protein expression levels of phospho- and total ERK and Akt in the presence and absence of melatonin in both CSCs and SKOV3 cells. Our results revealed a significant decrease in phospho- ERK expression in SKOV3 cells with a partial effect in CSCs when incubated the cells with melatonin (3.4 mM) (Fig. 5a). Akt protein expression analysis also showed a significant decrease in phospho- Akt protein level in SKOV3 cells with a relatively smaller change in that level in CSCs (Fig. 5a).

To study the effect of melatonin on migration of CSCs, transwell migration assay was employed. Our results revealed a markedly higher migration rate in CSCs compared to SKOV3 cells (Fig. 5b). Melatonin significantly repressed the migration of CSCs (p < 0.05) with only a slight decrease in this rate in SKOV3 cells (p > 0.05). To investigate whether melatonin inhibits migration of CSCs through MAPK or PI3K signaling pathway, the cells pre-incubated with PD098059 and LY294002 as MAPK and PI3K inhibitors, respectively. The results showed that inhibitory effect of melatonin on cell migration enhanced in the presence of both MAPK and PI3K inhibitors in SKOV3 cells, while inhibition of these pathways did not show any significant effect on migration of melatonin-treated CSCs (Fig. 5b). Our results suggest that melatonin-induced inhibition of migration in CSCs is carried out through different signaling pathways. Pre-treatment of the cells with luzindole revealed a partial abolishment in anti-migratory effects of melatonin on CSCs suggesting the partial contribution of receptor-independent effects of melatonin in migration process (Fig. 5b). Our results from both Western blot analysis and migration assay suggested the key role of ERK and MAPK signaling pathway in migration of SKOV3 cells but not CSCs.

## Discussion

The effects of melatonin on proliferation, invasion and metastasis of cancer cells have been recently described^8^. The main purpose of this study is to provide advanced insights into specific anti-tumor effects of melatonin on ovarian cancer stem cells. For this purpose, we first isolated CSCs based on CD133 and CD44 surface markers. Previous studies suggested CD133 and CD44 as the most frequent markers for isolation of prostate and ovarian cancer cells^14,15^. Our data revealed a CSCs subpopulation of 1.28% in SKOV3 cell line. This result is consistent with previous studies which showed a various populations of CSCs between 0.1–1.5% in cervical, melanoma, prostate and glioblastoma cancer lines^16^. To confirm the stemness of isolated cells, spheroid formation experiments revealed a marked increase in renewal ability of these cells along with a significant increase in the expression of SOX2. Other studies also applied spheroid formation assay and SOX2 expression for isolation and confirmation of stemness properties in CSCs^17,18^.

Next, we investigated the impact of melatonin on stemness, proliferation, invasion and migration of CSCs. Melatonin caused a marked decrease in the expression of SOX2 and Nanog. These molecules along with Oct4 regulate pluripotency and stemness in embryonic, adult and cancer stem cells^19,20^. More recently, it has been reported that overexpression of SOX2 in CSCs is related to the invasive behavior and aggressiveness of human ovarian carcinoma^21^. Melatonin also decreased SOX2 expression in glioblastoma CSCs^6^. We showed that melatonin decreased proliferation and induced apoptosis in both CSCs (IC50: 6.3 mM) and SKOV3 cells (IC50: 3.4 mM). This result is consistent with other study revealing a greater resistance in CSCs cells mostly because of higher expression and activity of ABC transporters^22^. We also determined a significant amount of Ki67 in CSCs which is consistent with the studies showing that Ki67 expression has been elevated in CD133^+^, CD44^+^/CD24^−^ and ALDH^+^ CSCs cells^23,24^. Melatonin also decreased Ki67 in CSCs from 85.23 to 67.07%. Recent studies have demonstrated that besides the role of Ki67 in cell proliferation, this molecule is also involved in metastasis and invasion of cancer cells^25,26^. Melatonin induced a slight decrease in proliferation rate in non-stem cell SKOV3 cells (p < 0.05). This discrepancy between cell viability and proliferation in SKOV3 cells can be explained by considering the induction of different apoptosis pathways by melatonin. Previous studies showed that melatonin could induce apoptosis in many cancer types^27^. In the study melatonin enhances apoptosis by increasing of p53 acetylation and modulating of MDM2/MDMX/p300 pathway^28^. However the concentration of melatonin differs from nano molar to mili molar based on type of cancer cells, incubation condition, duration of exposure and the number of cells in cell culture stocks^27^.

Melatonin functions through both receptor-dependent and independent pathways^9,10^. The expression of melatonin receptor, MT1 has been described in nervous system, kidney, gall bladder, mammary gland, skin and ovary, however there was a few information about MT2 expression in ovarian cancer^9,29^. According to our results, the mRNA expression levels of both receptors MT1 and MT2, have been higher in SKOV3 cells than those in CSCs while the protein expression of these receptors are the same in both of cell populations. Jablonska *et al*. determined that the expression of MT1 negatively correlated with malignancy grade and aggressiveness of breast cancer. They also suggested that MT1 expression level was directly proportional to the survival rate^30^. Desensitization, internalization and down regulation of MT1 and MT2 receptors are the main mechanisms of regulation of these receptors depends on the type of the cells, and concentration and exposure time of melatonin^31^. There are several controversial studies related to melatonin receptor regulation under melatonin treatment^9,32,33^. In the study performed by Carbajo-Pescador *et al*., melatonin increased melatonin receptor expression through elevating of mRNA and protein stability^9^. However, other studies displayed that melatonin decreased expression of its receptors in breast and colorectal cancer cells^32,34^. We showed that melatonin decreased both MT1 and MT2 expressions in mRNA level but not protein level in both CSCs and SKOV3 cancer cells. Similar data were also obtained on the effects of melatonin in endometrial cancer lines^33^. This observation can be explained by lower stability of melatonin receptors. In addition, luzindole, as an antagonist of melatonin receptors, could not completely inhibit anti-proliferative activity of melatonin. Carbajo-Pescador also reported that luzindole partially inhibited melatonin effects in hepatocarcinoma cancer cells^35^. These results suggested that melatonin functions partially through its specific receptors. However, other receptor-independent signaling pathways may be responsible for anti-tumor effects of melatonin.

EMT plays an essential role in tumor metastasis, migration and invasion, which is tightly related to the characteristics of CSCs^36^. The cancer cells shift from epithelial to mesenchymal phenotype during EMT process with changes in expression of EMT-related transcription factors and protein markers including ZEB1, ZEB2, snail, vimentin and E-cadherin^37^. We next investigated the role of melatonin in alteration of the expression in these markers in both CSCs and SKOV3 cells. We found attenuation in the expression of E-cadherin and elevation in the expression of vimentin and EMT transcription factors including snail, ZEB1 and ZEB2 in CSCs which is consistent with previous studies in colorectal and breast tumors^38,39^. Melatonin enhanced E-cadherin and decreased vimentin, snail, ZEB1 and ZEB2 expression levels in CSCs. Few studies have been published on the effect of melatonin on EMT process in CSCs. In accordance with our results, Gonçalves *et al*. reported that there were low expression of E-cadherin and high expression of N-cadherin and vimentin in isolated CSCs from human and canine breast cancer cell lines which were reversed by melatonin^13^. Another study demonstrated that melatonin elevated E-cadherin and diminished vimentin in the mice mammary tumor and MCF-7 cells by activation of GSK3β, induction of snail and degradation of β-catenin as an E-cadherin repressor^39^.

In addition to the EMT process, matrix metalloproteinase (MMPs) specially MMP-2 and MMP-9 also play an important role in invasion and metastasis^40^. There is accumulative evidence that MMP-2 and MMP-9 mediated poor prognosis of several solid tumors by degradation and remodeling of the extracellular matrix^36^. In this study, we assessed the impact of melatonin on activity and expression levels of MMP-2 and MMP-9 in both stem and non-stem ovarian cancer cells. There was a strong activity of MMP-9 but not MMP-2 in CSCs while gene expression levels of these MMPs were lower in CSCs compared to those in non-CSCs. This discrepancy between mRNA and activity levels of MMP9 can be explained by the impact of melatonin on degradation and post-transcriptional modifications of these proteins. Also it has been reported that nucleolin or TGF-β1 affects MMP-9 stability^41^. It can be concluded that in contrast to total RNA or protein levels, the activity of these molecules can be considered as more valuable parameters with tight association with stemness and aggressiveness of cancer cells. Previous studies suggested that the activity and expression of MMPs were related to the stemness properties^2,36^. MMP-2 and MMP-9 led to the up regulation of EMT-related proteins, Wnt signaling components and stemness properties^36^. Overexpression of MMP-9 attenuated E-cadherin protein level while silencing MMP-9 gene caused elevation in E-cadherin in ovarian cancer cells^42^. Furthermore, mesenchymal stem cells induced prostate cancer stem cell phenotype by promoting MMP-9 and snail expression and enhancing invasion^43^. We have also found that melatonin exerts an inhibitory effect on the activity and expression levels of MMP-2 and MMP-9 in both CSCs and non-CSCs. Previous studies demonstrated that melatonin reduced cell invasion by decreasing expression of MMP-2 and MMP-9 with an inhibitory effect through binding to MMP-9 active site^44,45^. Our data suggest that melatonin inhibits invasion of CSCs via down-regulation of EMT-related proteins and activity of MMP-9 but not MMP-2.

Our results revealed a significant anti-migratory effect of melatonin on CSCs with a slight inhibition on non-stem cells SKOV3 cells which was partially melatonin receptor dependent. Various studies have verified melatonin involvement in the prevention of tumor migration in various cancer cells but not in stem cell or non-stem cell ovarian cancer cells^8,34^. However, the precise mechanisms by which melatonin regulates ovarian cancer cell migration are not yet fully understood.

To determine the signaling pathways that are involved in melatonin function in the migration of ovarian cancer cells, we investigated MAPK and PI3K as key signaling pathways^46^. Our data revealed that melatonin exert anti-migratory effects by MAPK and PI3K pathways in ovarian cancer cells but not in isolated CSCs. Recent studies indicated that inhibitory effect of melatonin on proliferation, invasion and migration are mediated by down-regulation of the p38 MAPK pathway in breast and gastric cancers^12,47^. In ovarian cancer, it has been shown that melatonin in combination with cisplatin prevents phosphorylation of ERK1/2 and p90RSK^7^. Moreover, Gao *et al*. indicated that melatonin along with 5-FU decrease survival, migration and invasion abilities of colon cancer cells by targeting the PI3K/AKT and NF-κB signaling pathway^48^. However, very few studies were performed on underling mechanisms of melatonin dependent migration of CSCs. Our results indicated that melatonin inhibits migration in CSCs independent of MAPK and PI3K pathways. In contrast to our results, Chen *et al*. demonstrated that melatonin inhibits AKT activation and thereby prevents PI3K signaling activity in MT1 dependent manner in glioblastoma cancer stem cells^6^. Other study, showed that melatonin inhibits metastasis by modulating Rho-associated kinase protein-1 expression in breast cancer *in vitro* and *in vivo* ^49^. However, anti-migration and invasion effects of melatonin in gelioma cells induced via inhibition of ROS production and its downstream signaling, (ROS)/NF-κB/MMPs pathway^8^.

In conclusion, we first isolated cancer stem cells from SKOV3 cell line and characterized them for stemness properties. Then, we investigated the impact of melatonin in proliferation, migration and invasiveness of these cells. We showed that melatonin inhibited proliferation, migration, MMP-2 and 9 activities in a partial melatonin receptor dependent manner. Despite the key role of PI3K and MAPK pathways in SKOV3 cell migration, this phenomenon in CSCs was independent of both signaling pathways. The role of other signaling pathways including Rho-GTPase, NF-κB, ROS or GS3Kβ needs to be elucidated for CSCs. Melatonin also diminished EMT process in CSCs by down regulating of the genes that are involved in this phenomenon.

In summary, we suggest that identifying ovarian cancer patients with high melatonin receptor expression and then administration of melatonin, as an adjuvant for chemotherapeutic agents, can be considered as an important strategy to overcome tumor invasion and metastasis.

## Methods

### Cell culture and reagents

SKOV3 cell line (NCBI code: C209), was purchased from the National Cell Bank of Iran (NCBI), Pasteur Institute, and cultured in RPMI 1640 (Sigma-Aldrich, USA) medium supplemented with 10% v/v Fetal Bovine Serum (FBS; Gibco, USA), 100U/ml Penicillin and 100 μg/ml streptomycin (Gibco, USA) and maintained in a humidified atmosphere of 95% air and 5% CO_2_ at 37 °C.

### Melatonin preparation

Melatonin (Sigma-Aldrich, china) was dissolved in dimethyl sulfoxide (DMSO, Merck, Germany) to prepare 0.2 M (50 mg/ml) stock solution. Then, the stock solution was diluted with RPMI-1640 to prepare different concentrations of working solution immediately before use.

### Magnetic activated cell sorting assay

CD133^+^ cells were isolated and enriched from SKOV3 cell line using MACS assay according to the manufacturer’s instruction. Briefly, the adherent cells were detached with 2.5% trypsin-EDTA (Gibco, USA), washed and re-suspended in PBS. Fc receptors were blocked with FCR blocking reagent (Miltenyi Biotec., Germany) and then the cells (1 × 10^7^) were incubated with 20 µl of CD133 microbeads (Miltenyi Biotec., Germany) for 20 min on rotator at 4 °C. Subsequently, cells were washed with PBS containing 0.5% bovine serum albumin and enriched by MidiMACS magnet (Miltenyi Biotec., Germany) using LS, MACS manual cell separation columns (Miltenyi Biotec., Germany). CD133^+^ cells were collected as primary CSCs for further studies.

### Spheroid formation assay

Cells (3 × 10^4^) from single cell suspensions were cultured in a serum free medium containing DMEM/F12 (Gibco, UK) supplemented with 10 µM basic fibroblast growth factor (bFGF, Sigma-Aldrich, USA) and 10 µM epidermal growth factor (EGF, Sigma-Aldrich, USA) in non-treated 6 well culture plates (SPL, Korea). Culture media were replaced or supplemented with fresh growth factors every three days. After 7 days, each well was examined for formation of tumorsphere-like cell aggregates by an inverted microscope (Labomed, USA).

### Cell viability assay

The effect of melatonin on cell viability was determined by a colorimetric assay using (3-(4, 5-Dimethylthiazol-2-yl)-2,5-Diphenyltetrazolium Bromide (MTT) (Sigma-Aldrich, USA). Briefly, cells (1 × 10^4^/well) were seeded in 96-well plate and cultured in RPMI-1640 medium containing 10% FBS. After 24 h, each well was further treated with various concentrations of melatonin (1–10 mM) in the same medium supplemented with 2% FBS for 24 h, 48 h or 72 h. Then, 20 µl of MTT solution (5 mg/ml) was added to each well and incubated for the next 4 h. The culture medium was removed and replaced with 200 µl DMSO solution. Finally, the absorbance value was measured at 630 nm using a micro-plate reader (Biotek Instruments, USA). Cell viability (IC50) was determined for each agent by calculating the slope and intercept of different concentrations. The experiment was performed in triplicate, and repeated three times.

### Flow cytometric analysis

Cells were stained with the antibodies against surface markers including FITC- CD133/2 (Miltenyi Biotec, Germany), PE- CD44 (Miltenyi Biotec, Germany) and intracellular markers including FITC-SOX2 (e-bioscience, USA), FITC-Ki67 (e-bioscience, USA) following the manufacturer’s instructions. Briefly, SKOV3 cells and CSCs were incubated with 3.4 mM of melatonin for 48 h, trypsinized with 2.5% trypsin-EDTA (Gibco, USA), washed twice with PBS, and incubated with 10 µl of antibodies at room temperature for 30 min in the dark. Permeabilization with 0.1% Triton X-100/PBS for 1 min was performed before incubation with the intracellular markers. The cell populations were then characterized according to the surface markers using a FACS Calibur flow cytometer (BD Bioscience, USA). The data were collected and analyzed using Flowjo Software (Tree Star Software, USA). Nonspecific protein labeling was identified by appropriate isotype-matched antibodies.

### RNA isolation and real-time RT PCR

Total RNA was extracted using RNA X plus solution (CinnaGen, Iran) according to the manufacturer’s procedure. The extracted RNA was reverse-transcribed into cDNA by a reverse transcription Kit (Bioneer, Korea). Real-time RT PCR was performed using QuantiTect SYRB Green dye (TaKaRa, Japan) and Corbett Rotor-Gene™ 6000 HRM system. Real-time RT PCR primer sequences were listed in Table 1. The expression levels of each gene were analyzed by Pfaffl methods with normalization to housekeeping gene, Glyceraldehyde-3-Phosphate Dehydrogenase (GAPDH). All experiments were carried out in triplicate.

**Table 1:** Sequences of primers used for real-time PCR analysis.

Gene	Forward primer	Reverse primer
MT1	5′-CCTGCGTCCTCATCTTCACCATC	5′-CAGGTCTGCCACCGCTAAGCTC
MT2	5′-CCTCCTCCCTATCGCTGTCGTGT	5′-TCCGCAAGTCGCTGGGCTTC
MMP2	5′-CACATAGTGATGGTTCCCCTGTT	5′-CGGCCACTCAGTAGGTGTCTTT
MMP9	5′-ATTTCTGCCAGGACCGCTTCTAC	5′-ATCCGGCAAACTGGCTCCTTC
SOX2	5′-TTGCTGCCTCTTTAAGACTAGGA	5′-CTGGGGCTCAAACTTCTCTC
ZEB1	5′-CTGGAGAAAAGCCCTATCAATGT	5′-CTGTCTTCATCCTCTTCCCTTGT
ZEB2	5′-CAGCCATTACCCAGTTAAGA	5′-CCCGTGTGTAGCCATAAGA
Snail	5′-GAGTTTACCTTCCAGCAGCC	5′-CAGAGTCCCAGATGAGCATT
vimentin	5′-CAGATGCGTGAAATGGAAGAGAA	5′-TAGGTGGCAATCTCAATGTCAA
e-cadherin	5′-TGCCCAGAAAATGAAAAAGG	5′-GTGTATGTGGCAATGCGTTC
Nanog	5′-CTGTGATTTGTGGGCTAA	5′-TGTTTGCCTTTGGGACTGGT
GAPDH	5′-CAAGATCATCACCAATGCCT	5′-CCCATCACGCCACAGTTTCC

### Immunofluorescence staining

SKOV3 and isolated CSCs were seeded in chamber slides (SPL, Korea) and treated with or without IC50 concentration of melatonin (3.4 mM) for 48 h. Then, the cells fixed with paraformaldehyde 4%, washed twice with PBS and incubated with FITC-conjugated anti-human CD133/2 and PE-conjugated anti-human CD44 antibodies for 30 min at room temperature. The nuclei were stained with 4′,6-diamidino-2-phenylindole (DAPI) (Sigma-Aldrich, USA). Finally, the cells were examined using a fluorescent microscope (Olympus system, Japan).

### Protein extraction and Western blot analysis

Cells (1.5 × 10^6^) were lysed in a protein extraction buffer (25 mm HEPES, 1% Triton X-100, 2 mm EDTA, 0.1 m NaCl, 25 mm NaF, 1 mm Sodium Orthovanadate) containing protease cocktail inhibitor (Roche, Basel, Switzerland) for 30 min on ice. Cell lysates centrifuged at 12000 g for 20 min. Then, the supernatant collected and concentration of protein was determined with picodrop (Picodrop LTD, Cambridge, UK). The equal amount of protein (~100 µg) loaded at 12% SDS- Acrylamide gel followed by transferring to PVDF membrane. Skim milk (5%) in PBS was used to block the membrane and then incubated with phospho ERK, phospho Akt, ERK, Akt and β-actin antibodies (Santa Cruz Biotechnology, Santa Cruz, USA) overnight at 4 °C. HRP-conjugated antibody was applied as a secondary antibody (Santa Cruz Biotechnology, Santa Cruz, USA) for 1 hour at room temperature and finally protein detection was performed with ECL reagent. Density of each band was determined using imageJ software.

### SDS-PAGE gelatin zymography of MMP-2 and MMP-9

Gelatin-zymography was performed to determine the effect of melatonin on MMP-2 and MMP-9 activities on CSCs and SKOV3 cells as previously described^44^. The equal amount (~100 µg) of extracting protein of each sample was mixed with an equal volume of 2X sample buffer (0.125 M Tris-HCl (pH 6.8), 4% SDS, 20% glycerol, and 0.004% bromophenol blue and subjected to 12% SDS-polyacrylamide gel containing 1 mg/ml gelatin (Sigma-Aldrich, USA) under non-reducing conditions. Following electrophoresis, the gel was washed twice with 2.5% triton-X100 solutions for 1 h to remove SDS and then incubated in zymography buffer containing 40 mM Tris-HCl, pH 7.4, 0.2 M NaCl and 10 mM CaCl2 overnight at 37 °C. Gels were then stained with 0.1% Coomassie blue staining solution (Sigma-Aldrich, USA) and destained in 1% acetic acid solution. Clear regions on the gels demonstrated gelatinolytic activity and quantified using densitometry linked to the proper software (Lab Image; Germany).

### Transwell Migration assay

To investigate the role of melatonin on cell migration and underling signaling pathways, the transwell migration assay was performed. The cells were pretreated with 40 µM MAPK inhibitor, PD 098059 (Sigma-Aldrich, USA), 20 µM PI3K inhibitor, LY294002 (Sigma-Aldrich, USA) and 10 µM melatonin receptor inhibitor, luzindole (Sigma-Aldrich, USA) for 1 h. Cells (1.5 × 10^4^) were seeded in the upper chamber (8-μ m pore membrane) of 24-well Transwell chambers (SPL, Korea) in RPMI1640 medium supplemented with 2% FBS and then incubated with melatonin (3.4 mM) for 48 h. Chambers were incubated at 37 °C, and the cells were allowed to migrate through the pores in the filter for 48 h. Migrated cells to lower chamber were then counted in ten different fields. The results were calculated from three independent experiments.

### Statistical analysis

Data were expressed as mean ± standard deviation (SD) which resulted from three independent experiments. Statistical analysis of data was subsequently determined by t-test or ANOVA and Tukey (post hoc) test for multiple comparisons using GraphPad Prism software version 7.0 (GraphPad Software Inc., San Diego CA) and p value < 0.05 was considered as statistically significant.

## Electronic supplementary material

supplementary information
