# Supplementary material for: The potential therapeutic effect of melatonin on human ovarian cancer by inhibition of invasion and migration of cancer stem cells
Source: Sci Rep. 2017 Dec 6;7:17062. doi: 10.1038/s41598-017-16940-y (PMC5719004; doi:10.1038/s41598-017-16940-y)
Supplement: Supplementary file 1 — supplementary information [file 41598_2017_16940_MOESM1_ESM.pdf]

The potential therapeutic effect of melatonin on human ovarian cancer by inhibition of invasion and migration of cancer stem cells

Maryam Akbarzadeh [1, 2]. Ali Akbar Movassaghpour[3]. Hossein Ghanbari[4]. Maryam Kheirandish[5]. Nazila Fathi Maroufi [6]. Reza Rahbarghazi [7]. Mohammad Nouri [2, 1*] and Nasser Samadi [2, 6*]

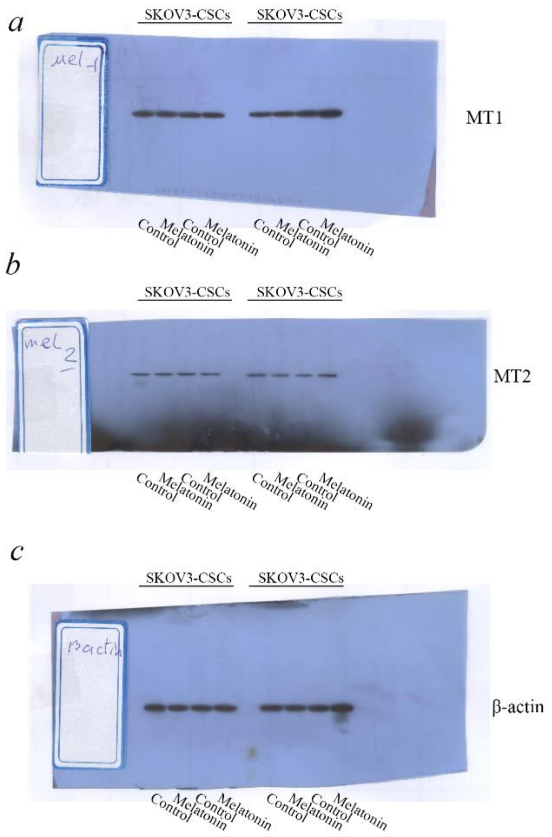

Figure 1. SKOV3 and isolated CSCs were treated with the 3.4 mM melatonin for 48 hr. The cell lysates were prepared and used for Western blot with MT1, MT2 and b-actin antibodies. The figure shows (***a***) melatonin receptor 1, (***b***) melatonin receptor 2 (***c***) beta actin in both SKOV3 and cancer stem cells. The experiment was repeated twice and the gel was not cropped.

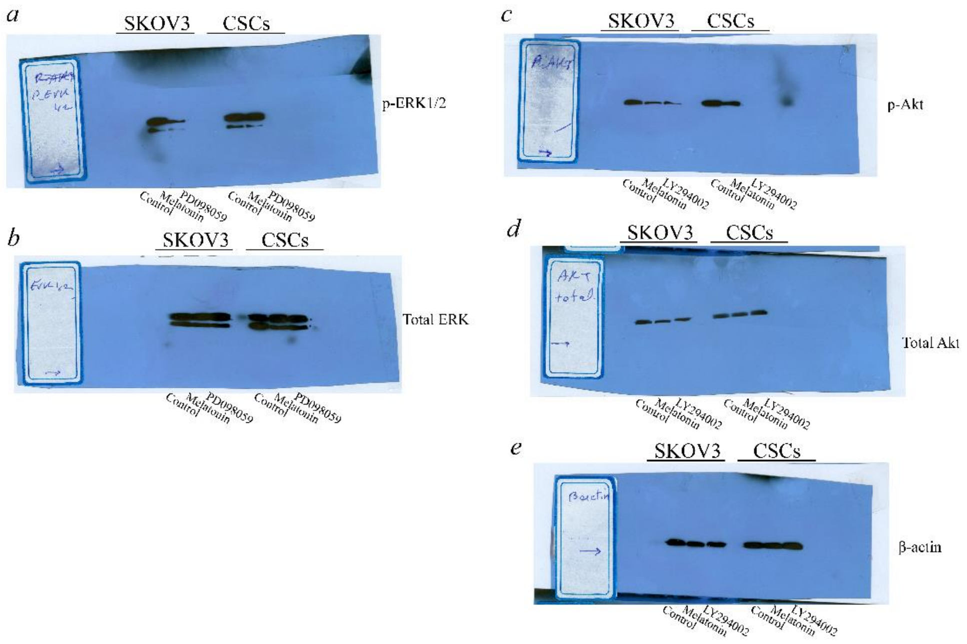

Figure 2. SKOV3 and isolated CSCs were treated with the 3.4 mM melatonin for 48 hr. The cell lysates were prepared and used for Western blot with (***a***) p-ERK1/2, (***b***) total ERK, (***c***) p-Akt, (***d***) total Akt and (***e***) b-actin antibodies in both SKOV3 and cancer stem cells. the gel was not cropped.
